# Human factors in escalating acute ward care: a qualitative evidence synthesis

**DOI:** 10.1136/bmjoq-2020-001145

**Published:** 2021-02-26

**Authors:** Jody Ede, Tatjana Petrinic, Verity Westgate, Julie Darbyshire, Ruth Endacott, Peter J Watkinson

**Affiliations:** 1 Adult Intensive Care Unit, Oxford University Hospitals NHS Foundation Trust, Oxford, UK; 2 Plymouth University, Plymouth, UK; 3 Bodleian Health Care Libraries, University of Oxford, Oxford, UK; 4 Nuffield Department of Clinical Neurosciences, University of Oxford, Oxford, Oxfordshire, UK; 5 School of Nursing & Midwifery, Monash University, Clayton, Victoria, Australia

**Keywords:** continuous quality improvement, critical care, decision making, human factors, medical education

## Abstract

**Background:**

Identifying how human factors affect clinical staff recognition and managment of the deteriorating ward patient may inform process improvements. We systematically reviewed the literature to identify (1) how human factors affect ward care escalation (2) gaps in the current literature and (3) critique literature methodologies.

**Methods:**

We undertook a Qualitative Evidence Synthesis of care escalation studies. We searched MEDLINE, EMBASE and CINHAL from inception to September 2019. We used the Critical Appraisal Skills Programme and the Grading of Recommendations Assessment-Development and Evaluation and Confidence in Evidence from Reviews of Qualitative Research tool to assess study quality.

**Results:**

Our search identified 24 studies meeting the inclusion criteria. Confidence in findings was moderate (20 studies) to high (4 studies). In 16 studies, the ability to recognise changes in the patient’s condition (soft signals), including skin colour/temperature, respiratory pattern, blood loss, personality change, patient complaint and fatigue, improved the ability to escalate patients. Soft signals were detected through patient assessment (looking/listening/feeling) and not Early Warning Scores (eight studies). In contrast, 13 studies found a high workload and low staffing levels reduced staff’s ability to detect patient deterioration and escalate care. In eight studies quantifiable deterioration evidence (Early Warning Scores) facilitated escalation communication, particularly when referrer/referee were unfamiliar. Conversely, escalating concerning non-triggering patients was challenging but achieved by some clinical staff (three studies). Team decision making facilitated the clinical escalation (six studies).

**Conclusions:**

Early Warning Scores have clinical benefits but can sometimes impede escalation in patients not meeting the threshold. Staff use other factors (soft signals) not captured in Early Warning Scores to escalate care. The literature supports strategies that improve the escalation process such as good patient assessment skills.

**PROSPERO registration number:**

CRD42018104745.

## Introduction

### Failure to rescue

‘Failure to rescue’ (FTR), defined as mortality following complications during a hospital admission,^1^ is common.^2^ At least 11 000 hospital patients each year suffer preventable deaths^3^ though other sources believe this number to be higher.^4^ It is also recognised that patients who die following a cardiac arrest are likely to have preceding warning signs that are not adequately managed.^5^ Though differences between hospital complication rates are small, patients can be three times more likely to die from complications depending on which hospital they are in.^2^ Poor surveillance of these patients can be linked to inadequate monitoring of abnormal vital signs, poor fluid balance management or diagnostic errors.^3 6^ Reports to the National Reporting and Learning System deomstrate that 7% were related to a failure to act or recognise patient deterioration.^7^

### Escalation of care

Avoiding FTR requires successful escalation of care^8^ whereby patients’ deteriorations are detected, communicated and acted on.^8 9^ Escalation interventions focus primarily on specialist clinical teams such as Critical Care Outreach or Rapid Response Teams (RRT).^10^ These teams aim to target improvements to the initial detection and ward management of patient deterioration.^11^ Other interventions target communication breakdowns.^12^

Human factors (HF) identified to positively or negatively affect care escalation include situational awareness, team working, communication, safety culture, workload, clinical experience, negative emotions and leadership.^6 9 13–15^ However, research has historically focused on outcomes.^8^ The aims of this qualitative evidence synthesis (QES) are to identify (1) how HF affect ward care escalation (2) gaps in the current literature and (3) critique literature methodologies.

## Methods

The Preferred Reporting Items for Systematic Reviews and Meta-Analyses (PRISMA) guideline was adhered to^16^ (see online supplemental file 1). We undertook a QES of the literature exploring escalation of care. The research question was developed by the two authors (JE and VW) using the Population, Interest and Context framework.^17^ A full protocol has been published in a peer-reviewed journal.^18^

10.1136/bmjoq-2020-001145.supp1 Supplementary data

The search strategy was assisted by a specialist librarian (TP). Searches were performed on three databases, MEDLINE, EMBASE and CINHAL. Dates searched were from database inception to September 2019. Medical Subject Headings terms were used and searched as free text (full search strategy is included in online supplemental file 2). Reference lists of all eligible studies were also checked, and incidental references included from these.

10.1136/bmjoq-2020-001145.supp2 Supplementary data

### Eligibility criteria

This evidence synthesis includes qualitative studies reporting primary data. No limits on publication date or country were applied. We included studies that explored how HF affect FTR and care escalation from staff, patients or relative’s perspective. Qualitative methods include (but are not limited to) ethnography, interviews, focus groups and HF methods. We defined HF as any human interaction affecting teamwork, tasks, equipment, workspace, culture or organisation.^19^ Data analysis included, but has not been not limited to, thematic analysis, grounded theory and discourse analysis.

#### Inclusion

- Qualitative studies reporting primary data.
- Qualitative studies exploring how HF affect escalation of care of the in-hospital patient population.
- Studies employing qualitative data collection methods, for example, semistructured interviews, focus groups or observations.
- Observational studies relating to FTR or care escalation.
- Adult population.

#### Exclusion

- Systematic or literature reviews.
- Correspondence and short communications.
- Simulation studies.
- Studies written in any language other than English.
- Studies in the emergency department and maternity.

Eligible studies were entered into Covidence systematic review software (Veritas Health Innovation, Melbourne, Australia. Available at www.covidence.org) and deduplicated. Study screening and selection was undertaken by two reviewers independently. The titles and abstracts were screened against the eligibility criteria. Disagreements between reviewers were resolved by third person mediation. Reasons for excluding studies were noted.

### Quality assessment and confidence in synthesised findings (Critical Appraisal Skills Programme and Grading of Recommendations Assessment, Development and Evaluation-CERQual)

Two researchers (JE and VW) reached a consensus regarding which study quality assessment tools to use during the review. Two different quality assessments were conducted on all studies by both researchers. The Critical Appraisal Skills Programme (CASP) qualitative checklist was used to assess papers for credibility, confirmability, dependability and transferability.^20^ This comprehensive framework tool is commonly used in qualitative study assessment.^21 22^

We assessed confidence in synthesised findings using the Grading of Recommendations Assessment-Development and Evaluation and Confidence in Evidence from Reviews of Qualitative Research (GRADE-CERQual) Criteria and associated guidance publications.^23–27^ The four-stage assessment (methodological limitations, coherence, adequacy of data and relevance) examines each synthesised finding for confidence by critiquing contributing study rigour.^28^ The output of this evaluation is a Summary of Qualitative Findings table detailing themes and papers contributing to this theme. This table promotes transparency in the synthesis methods. Themes from the data analysis are presented in order of highest to lowest confidence according to the GRADE-CERQual assessment.

### Analysis

We undertook a thematic synthesis^22^ using Thomas and Harden’s framework to map how HF affect escalation of care.^29^ This is a three-stage process. Initially, study findings are coded, these codes are then categorised into descriptive themes and finally these descriptive themes are categorised into analytical themes.^30^ Stage 1 involves line-by-line coding of data, where each sentence is allocated a code. Stage 2 involves categorising each coded sentence into descriptive, broader themes. The final stage involves generating analytical themes, or ‘going beyond’ the findings of the initial study, which relate to the fixed or emerging research question (see table 1 for definitions of analytical themes). This framework supports data extraction from anywhere within the paper and is not confined to the results alone.

**Table 1:** Definitions of analytical themes

Analytical theme	Definitions and references
Information packaging	The use of quantifiable evidence of deterioration (such as vital signs) to initiate escalation of care.^15 33 34 40 41^
Flattened hierarchy	Escalation of care can be initiated from any staff member to any staff member.^15 31 35 41–45^
Situational awareness	The comprehension of clinical elements and projection of their status in the future.^73^
Team functioning	Fragmented team-working with sequential rather than concurrent task completion and poor relationships.^35 36 41–45 47 48^
Soft signals of deterioration	Non-numerical deterioration cues attained from observation rather than instrumentation.^15 31 35–37 41 49 50 54 55^
Decision making	Clinical reasoning surrounding detection, communication and management of escalation of care.
Clinical experience	As staff became familiar with deteriorating patients, they were better able to detect and predict impending illness.^15 36 38 42 43 46 49 50 54 55^
Clinical assessment	Involves staff looking, listening and feeling the patient to identify respiratory, skin, neurological or physiological abnormalities.^15 49 51 55 57^

Data extraction tools were developed and piloted before the review took place to ensure consistency of data extraction. Study data were entered into an Excel spreadsheet (Windows, 2019. Microsoft Office) and study themes were analysed using NVivo software (NVivo qualitative data analysis Software; QSR International, V.10, 2014).

### Patient and public involvement

Patient representative (TD) reviewed the original published protocol and aims of the review were discussed and deemed of patient importance.

## Results

The search identified 2404 papers which met the initial search criteria (refer to online supplemental file 3 for PRISMA diagram). After duplicates were removed, 1651 articles were screened. 1627 were excluded based on methodology, subject of interest or incorrect population. This resulted in 24 papers meeting the inclusion criteria and being reviewed in full. A full description of synthesised study characteristics are presented in table 2.

10.1136/bmjoq-2020-001145.supp3 Supplementary data

**Table 2:** Synthesised studies summary table

Study ID	Study design	Name of journal	Methods	Sample size (N/hours)	Population	Data collection date	Data analysis method
Andrews^33^ 2005	Qualitative Design	Issues and Innovation in Nursing Practice	Interviews Observations	44 Not avail	Nurses, doctors, AHPs and CSWs	2002	Grounded Theory
Astroth^45^ 2013	Qualitative Design	Journal of Clinical Nursing	Interviews	15	Nurses	Not avail	Concept Analysis
Braaten^51^ 2015	Descriptive Qualitative	American Journal of Nursing	Interviews	12	Nurses	2012	Content Analysis
Brady^42^ 2014	Qualitative Design	BMJ Quality and Safety	Focus Groups	31	Nurses, respiratory therapists, physicians	2009	Constant comparison
Bunkenbor^55^ 2013	Descriptive Qualitative	Journal of Advanced Nursing	Interviews Observations	13, 70 hours	Nurses	2009	Content Analysis
Burns^46^ 2017	Qualitative Design	Journal of Advanced Nursing	Interviews	25	Nurses	2015	Thematic Analysis
Chua^49^ 2013	Qualitative Exploratory Descriptive	International Nursing Review	Interviews	15	Nurses	2011	Content Analysis
Chua^52^ 2019	Qualitative Exploratory Descriptive	International Journal of Nursing Studies	Interviews	22	Nurses	2016–17	Thematic Analysis
Currey^32^ 2017	Descriptive exploratory	Australian Critical Care	Survey	207	Nurses, Doctors, Care Support Workers,	2014	Content Analysis
Donohue^15^ 2010	Qualitative Design	Intensive and Critical Care Nursing	Interviews	9	Nurses	2006	Thematic Analysis
Elmufudi^43^ 2017	Qualitative Design	American Journal of Medical Quality	Interviews	40	Doctors	2014	Thematic Analysis
Foley^56^ 2018	Qualitative Design	Journal of Clinical Nursing	Interviews Observations	8	Nurses, CSWs		Systematic Text Condensation
Hart^40^ 2016	Descriptive Qualitative	Journal of Clinical Nursing	Interviews	28	Nurses	2015	Constant Comparison
Mohammmed Iddrisu^54^ 2018	Qualitative Design	Journal of Clinical Nursing	Focus Groups	14	Nurses	2014	Thematic Analysis
James*l*^31^ 2010	Qualitative Design	Journal of Nursing Management	Survey	131	Care Support Workers	Not avail	Content Analysis
Johnston^8^ 2015	Qualitative Design	Annals of Surgery	Observations	42 hours	Surgical wards	2013	Grounded theory
King 2019^72^	Qualitative Design	Health Expectations	Focus Groups	26	Patients and families	2014–2017	Thematic Analysis
Searle Leach^38^ 2010	Qualitative Design	Quality and Safety Healthcare	Interviews	50	Nurses	Not avail	Grounded Theory
Mackintosh^34^ 2012	Comparative case study	Postgraduate Medicine Journal	Interviews Observations	35 150 hours	Doctors, nurses, critical care nurses,	2009	Thematic Analysis
Mackintosh^41^ 2014	Qualitative Design	Social Science and Medicine	Interviews Observations	35 180 hours	Doctors, nurses, critical care nurses,	2009	Thematic Analysis
Massey^44^ 2014	Qualitative Design	Australian Critical Care	Interviews	15	Nurses	2011	Thematic Analysis
Martland^47^ 2016	Qualitative Design	Australian Health Review	Focus Groups	43	Doctors and nurses	2007	Grounded Theory
Peebles^48^ 2012	Service evaluation	Resuscitation	Observationsnotes review	17 care episodes	RRT episodes	Not avail	Thematic Analysis
Petersen^50^ 2017	Qualitative Design	BioMedical Central	Focus Groups	18	Nurse	Not avail	Content Analysis

RRT, rapid response team.

### Quality assessment results

#### Critical Appraisal Skills Programme

Studies were assessed to be of moderate to high quality and no studies were excluded based on this assessment (table 3). Two studies^31 32^ used surveys to understand nurses’ perceptions of caring for deteriorating patients and were scored poorly for choice of methodology. These studies were still included as open ended free-text questions were used and it was felt that this could still contribute to answering the research question, while acknowledging data from these studies are unlikely to be rich and is therefore a limitation of the method.

**Table 3:** CASP quality assessment for synthesised studies

Study ID	Was there a clear statement of the aims of the research?	Was the research design appropriate to address the aims of the research?	Was the recruitment strategy appropriate to the aims of the research?	Was the data collected in a way that addresses the research issue?	Was the data analysis sufficiently rigorous?	Is there a clear statement of findings?	Is a qualitative methodology appropriate?	How valuable is the research?	Have ethical issues been taken into consideration?	Has the relationship between researcher and participants been adequately considered?
Andrews^33^ 2005	Low	Low	Unclear	Low	Low	Unclear	Low	Low	Low	high
Astroth^45^ 2013	Low	Low	Low	Low	Low	Low	Low	Low	Low	high
Braaten^51^ 2015	Low	Low	Low	Low	Low	Low	Low	Low	Low	high
Brady^42^ 2014	Low	Low	Low	Low	Low	Unclear	Low	Low	Low	high
Bunkenborg^55^ 2013	Low	Unclear	High	Low	Unclear	Low	Low	Low	Low	high
Burns^46^ 2017	Low	Unclear	Unclear	Unclear	Low	Unclear	Low	Low	Low	high
Chua^49^ 2013	Low	Low	Low	Low	Low	Low	Low	Low	Unclear	high
Chua^52^ 2019	Low	Low	Low	Low	Low	Low	Low	Low	Low	low
Currey^32^ 2017	Low	Low	Low	Unclear	Unclear	Unclear	Low	Low	Low	high
Donohue^15^ 2010	Low	Low	Low	Low	Low	Low	Low	Low	Unclear	Unclear
Elmufdi^43^ 2017	Low	Low	Low	Low	Low	High	Low	Unclear	Low	high
Foley^56^ 2018	Low	Low	Low	Low	Low	Low	Low	Low	Low	high
Hart^40^ 2016	Low	Low	Low	Low	Low	Low	Low	Low	Low	low
Iddrisu^54^ 2018	Low	Low	Unclear	Low	Low	Low	Low	Low	Low	high
James^31^ 2010	Low	Low	Low	Low	Low	Low	Low	Low	Low	high
Johnston^8^ 2015	Low	Unclear	Low	Low	Low	Low	Low	Low	Low	high
King 2019^72^	Low	Low	Low	Low	Low	Low	Low	Low	Low	High
Leach^38^ 2010	Low	Low	Unclear	Low	Low	Low	Low	Low	Low	high
Mackintosh^34^ 2012	Low	Low	Low	Low	Low	Low	Low	Low	Low	high
Mackintosh^41^ 2014	Low	Low	Low	Low	Low	Low	Low	High	Low	high
Martland^47^ 2016	Low	Low	Unclear	Low	High	Low	Low	Low	Low	high
Massey^44^ 2014	Low	Low	Low	Low	Unclear	Low	Low	Low	Low	low
Peebles^48^ 2012	Low	Low	Low	Low	Unclear	Low	Low	Low	Unclear	high
Petersen^50^ 2017	Low	Low	High	High	Low	Low	Low	Low	Low	high

CASP, Critical Appraisal Skills Programme.

#### Grading of Recommendations Assessment, Development and Evaluation-CERQual

Following the CASP assessment all studies were evaluated against the GRADE-CERQual criteria. A Summary of Qualitative Findings table (table 4) is presented which promotes transparency in this synthesis’ findings and methods. The table includes documented rationale for grading judgements.

**Table 4:** Confidence in synthesised findings using the GRADE-CERQual framework

Summary of review finding	Studies contributing to the finding	Methodological limitations	Coherence	Adequacy	Relevance	CERQual confidence assessment	Explanation of the CERQual evidence
**Information packaging** (using quantifiable evidence of patient deterioration) affected perceived **communication credibility**	15 33–39	Low concerns regarding study methodology	Low concerns about coherence	Low concerns about adequacy	Low concerns about relevance	**High** Confidence	All studies, demonstrated good methodology, data were considered moderately thick with high numbers of participants and methods, a high no of studies contributed to review finding,
**Flattened hierarchy** and were organisational components affecting escalation of care	15 31 32 34 35 41–46	Low concerns regarding methodology	Low concerns about coherence	Low concerns about adequacy	Low concerns about relevance	**High** Confidence	One study with minor concerns regarding methodology (survey), high no of studies contributing to finding, data were considered moderately thick with high numbers of participants and methods
**Workload** and **staffing** were factors considered by clinical staff to affect their **Situational awareness** of patient deterioration.	15 32 34–36 41 42 45 47–52	Minor concerns regarding methodology	Low concerns about coherence	Low concerns about adequacy	Low concerns about relevance	**High** confidence	Two studies with minor methodological concerns with one study where using a survey, and another study using participants for a focus group put forward by head nurse, high no of studies contributing to review finding, rich data sources and multiple methods of data collection, data were considered moderately thick with high numbers of participants and methods
**Team functioning caused problems or facilitated care during escalation**	15 32 33 35 36 41–45 47–51	Minor concerns regarding methodology	Low concerns about coherence	Low concerns about adequacy	Low concerns about relevance	**High** confidence	Two studies with methodological concerns, one study where using a survey, and another study using participants for a focus group put forward by head nurse, all other studies demonstrate good methodology, high no of studies contributing to review finding, data were considered moderately thick with high numbers of participants and methods
**Soft signal of patient deterioration** used by clinical staff indicating a patient’s worsening condition, not adequately represented in **Early Warning Score**	15 31 33 35 36 38 41 42 44 46 49–51 54–56	Moderate concerns regarding methodology	Low concerns about coherence	Moderate concerns about adequacy	Low concerns about relevance	**Moderate** Confidence	Three studies had methodological concerns. One utilising a survey methodology with open ended-questions, the other was being observed by the implementer of the local Medical Emergency Team (MET), the last one using participants for a focus group put forward by head nurse, large no of studies contributing to synthesis finding,
Clinician confidence affected **decision making** during escalation of care	31–36 38 43–45 47 49–52 55	Moderate concerns about methodology	Low concerns about coherence	Moderate concerns about adequacy	Low concerns about relevance	**Moderate** confidence	Four studies had methodological concerns, two studies utilised a survey methodology with open ended-questions, the other study had a focus group where participants were selected by head nurse, the other had observation completed by the implementer of the local RRT, data were considered moderately thick with high numbers of participants and different methods, large no of studies contributing to synthesis finding
**Clinical Assessment skills** relating to **patient assessment** and staff experience positively or negatively affected deterioration detection by clinical staff	15 35 36 38 42 43 46 49–51 54 55	Moderate concerns regarding methodology	Low concerns about coherence	Moderate concerns about adequacy	Low concerns about relevance	**Moderate** Confidence	Two studies with methodological concerns. One had observations completed by the implementer of the local RRT, the other study had a focus group where participants were selected by head nurse, data were considered moderately thick

GRADE, Grading of Recommendations Assessment, Development and Evaluation; RRT, rapid response ream.

## Theme results

Themes presented are ranked from the highest to lowest confidence in synthesised findings. Data extracted mostly related to organisational and patient assessment factors affecting escalation of care. Organisational factors could be classified into Information Packaging and Communication Credibility, Flattened Hierarchy, Workload, Staffing and Situational Awareness and Team Functioning. We found patient assessment Themes of ‘Soft Signals of Patient Deterioration’ and Early Warning Scores (EWS), Decision Making and Clinical Assessment Skills and Experience.

### Information packaging and communication credibility (high GRADE-CERQual evidence)

Eight studies identified that information packaging during escalation of care was a facilitator to success.^15 33–39^ Packaging involved using quantifiable evidence of patient deterioration such as vital signs^15 33 34 40 41^ to initiate escalation of care. This removed ambiguity,^33^ provided numerical evidence of deterioration^15^ and was a common language^34^ for clinical staff. This was particularly important when staff were unfamiliar to each other.^34^ Conversely, staff felt communication credibility was questioned when referrals were made using non-medical language^33^ or delivered in an unsystematic way.^34^ This made an escalation referral difficult to understand and prioritise, with medics often having to question further to gain more information to facilitate decision making.^33^

### Flattened hierarchy (high GRADE-CERQual evidence)

A common organisational facilitator to escalation of care was a flattened hierarchy meaning that escalation is accepted from anyone to anyone.^15 31 35 41–45^ This created a confidence in staff to raise concerns regarding a patient’s clinical condition, opening channels of communication. Staff also felt that electronic vital signs systems increased the accountability of patient illness^32 35 42 46^ with acutely unwell patients being everyone’s responsibility. However, it was also acknowledged identifying who is accountable for an unwell patient was sometime a challenging. Synthesised studies demonstrated instances of lack of deteriorating patient ownership^32^ or passing on of the problem^34^ by clinical staff to another team or colleague.

### Workload, staffing and situational awareness (high GRADE-CERQual evidence)

Several studies described resources as a significant factor affecting care escalation. Three studies identified a lack of skilled staff as limiting the ability to escalate the deteriorating patient.^15 35 47^ During high workload or low staffing periods staff felt their awareness of patient deterioration reduced due to sensory overload and suboptimal monitoring due to competing demands.^15 32 34 36 41 42 45 47–52^ Staff believed continuity of care improved situational awareness.^36 41 42 49 51^ Staff felt that a benchmark ‘baseline’, meant they could identify any significant changes to patient illness. It was not uncommon for staff to employ workarounds during the periods of system pressure such as escalating to the RRT. This was done (rightly or wrongly) to supplement care escalation when medical support was scarce.^15 47^

### Team functioning (high GRADE-CERQual evidence)

Seven studies found poor team relationships were a barrier to escalating patient care, resulting in significant delays.^15 32 33 35 42 45 50^ Poor team working was presented as tasks being done sequentially rather than concurrently, or where there was a lack of role definition.^35 36 41–45 47 48^ Staff believed a lack of understanding of team roles and the care individuals could provide contributed to uncertainty about to whom patients should be escalated.^35 41 44 45^ Poor team functioning meant staff felt deterred from escalating care due to negative emotions such as fear of reprimand, fear of being wrong, intimidation and retribution.^32 35 42 45 49–51 53^ Escalation to outside resources, such as the RRT, was sometimes perceived to be negative^15 43 45^ with staff reporting that they preferred to cope with a patient deterioration.

### Soft signals of patient deterioration and EWS (moderate GRADE-CERQual evidence)

Staff at times overruled the EWS derived escalation pathways using other patient related factors in their decision-making process when considering escalation.^15 31 33 35 36 38 41 42 44 46 49–51 54–56^ They identified factors additional to standard EWS variables which caused them concern about a patient’s condition (see online supplemental file 4). These patient factors or ‘soft signals of deterioration’, were (from most to least common finding in studies); pale skin,^15 31 41 49 50^ respiratory pattern (as distinct from respiratory rate),^15 35 37^ blood loss,^36 49^ personality change,^36 49^ patient complaint,^50 54^ skin temperature^55^ and patient fatigue (observed or reported).^15^ Nine studies found patient assessment was integral to detecting the ‘soft signals of deterioration’ including the early signs of worsening illness before a triggering EWS was evident.^15 35 36 38 39 46 49 50 55^ Two papers described how staff felt that EWS protocols could place barriers to escalation when patients did not meet the trigger threshold but nurses felt they required an increase in care surveillance.^33 50^ In some instances staff felt they had to wait for a deterioration to occur before being able to escalate^33^ but in others they continued to escalate despite normal EWS.^46 49 51^

10.1136/bmjoq-2020-001145.supp4 Supplementary data

### Decision making (moderate GRADE-CERQual evidence)

Escalation decision making involved clinical reasoning surrounding the detection, communication and management of escalation of care. Seven papers found that clinician confidence is a facilitator to decision making during patient deterioration management.^31–33 35 36 44 50^ Confidence can be derived from staff providing peer support to one another, training or education level. Shared team decisions were sometimes an escalation facilitator.^38 44 45 47 49 51^ However, a lack of consensus in decision making particularly for end of life care,^32^ was seen as problematic^34 43 49 55^ often leading to deviation from guidelines or escalation protocols. Lack of consistency in decisions meant escalation of care demonstrated response variability,^34^ leading to differing and unpredictable priorities.^47^ There was also evidence of clinicians assuming physiology changes were not significant and waiting for confirmation of deterioration before responding meaningfully.^43^

### Clinical assessment skills and clinical experience (moderate GRADE-CERQual evidence)

Clinical assessment involved looking, listening and touching the patient to identify respiratory, skin, neurological or physiological abnormalities.^15 49 51 55 57^ The ability to clinically assess patients well enabled staff to make better escalation of care decisions,^35 36 38 43 49–51 55^ particularly as the ability to detect ‘soft signs’ was seen as key. Conversely, undertaking a poor patient clinical assessment posed barriers to illness detection.^15^ Many studies found that as staff gained experience of deteriorating patients they were better able to detect and predict impending illness.^15 36 38 42 43 46 49 50 54 55^

## Discussion

We identified 24 qualitative studies of moderate to high methodological quality that identify how HF affect escalation of care. Our evidence synthesis has contributed to escalation of care literature and themes derived from analysis are pertinent to clinical practice.

The studies within this synthesis demonstrated that EWS provide staff with a tool that facilitates communication of concerns and assists workload prioritisation.^33^ Studies reported successful escalation of care was best facilitated when a patient’s deterioration packaged neatly with quantifiable evidence. However, some staff in the synthesised studies felt able to escalate non-triggering patients requiring medical attention, although this process was acknowledged to be challenging. It was also suggested that some staff can anticipate clinical deterioration before a triggering EWS^58^ and that there are soft signals (fatigue, skin temp/colour, patient complaint, personality change, blood loss, respiratory pattern), of deterioration recognised by nurses but are not adequately captured by EWS in their current format. Many studies also found that as staff gained experience of deteriorating patients, they were better able to predict deterioration patterns and anticipate problems. It seems that the EWS alone may not maximise improvements to patient outcomes.^59 60^ Evidence suggests that organisations should facilitate good patient assessment, as this was key to detecting soft signals that would otherwise go undetected through an alerting system. Research should also aim to identify how clinical staff anticipate problems in certain patient groups and how they recognise and respond to these to ultimately create safety.^61^ It is evident that the literature does not fully report good escalation catches^62^ such as rescued non-triggering sick patients. This event is in effect invisible and not measured in current healthcare evaluation systems or metrics. Incorporating this tacit knowledge into education programmes or simulation training and scenarios, may be a feasible strategy to improve care escalation.

A flattened hierarchy^63^ was implemented in the aviation industry when it was discovered that a number of flight incidents may have been avoided had the copilot been empowered to challenge the pilot.^64^ Synthesised studies identified that a Flattened Hierarchy was felt by healthcare staff to be a positive strategy for escalating care of deteriorating patients (escalation can be initiated from anyone). However, the effectiveness of a flattened hierarchy may be influenced by poor team functioning. Poor team working was a common barrier to escalation of care identified in this evidence synthesis. This finding is corroborated by a retrospective case records review of preventable hospital deaths^3^ and a literature review on FTR following surgery.^65^ In both publications, the authors isolated several contributory factors such as poor team communication, leadership and decision making. Without adequate team communication, the benefits of a flattened hierarchy and team decision making may be lost. If organisations wish to implement a flattened hierarchy escalation system this must also be complemented with an emphasis on non-technical skills and training^66^ before evidence of full patient benefit.

A clinically significant theme to emerge from the synthesised findings was that the greater the workload, the less staff felt they were able to keep track of patient illness or monitor their patients. This sometimes resulted in staff undermonitoring their patients causing some triggering patient deterioration to go unnoticed.^50^ This finding is supported by a recent study demonstrating that lower numbers of registered nurses led to a higher rate of missed vital signs observation.^67^ Organisations could focus on reducing workload, (an unlikely solution), or improving vital signs monitoring processes. A recent option is utilising wearable continuous monitoring that may reduce the nursing workload spent performing regular vital signs observation rounds.^68^

Other significant clinical effects of high workloads may be a reduction in staff ability to detect deterioration in patients who are not triggering, losing the human safety net for false negative (non-triggering) patients. When mental capacity is limited with reduced team resources, this will directly affect an individual’s situational awareness of the environment as mental resources reduce as cognitive demands increase.^69^ A recent study found the risk of death increased by 3% for every day a patient experienced nurse staffing levels below ward mean.^70^ Poor situational awareness, reduced ability to detect soft signals of deterioration and undermonitoring may explain these results. Conversely, staff described improved situational awareness when there was continuity to their patient care. This was felt to facilitate staff in detecting often nuanced clinical changes or soft signals and also bridged the care elements through a patients illness.^71^ A strong local emphasis on nursing continuity should be encouraged as the evidence suggests that this may improve detection of deterioration and care escalation.

Our study has some limitations. Synthesised studies were assessed for their methodological robustness using the GRADE-CERqual and CASP guidelines. This enabled us to present themes with the highest confidence of good quality evidence first, but results may be limited by the data quality or analysis. within the studies themselves. Publication bis may also affect results that were included. Broadly, studies were methodologically sound but consistently failed to explore the relationship of the researcher to the participants, or this was not explicitly documented. There was also only one study identified that used patients and relatives as study participants.^72^

### Conclusion

This evidence synthesis has identified HF that affect escalation of care. EWS have clinical benefits but can sometime impede escalation in patients not meeting the escalation threshold. Staff use other factors (soft signals) not captured in EWS to escalate care. The literature supports strategies that improve the escalation process such as good patient assessment skills. An organisational emphasis on non-technical skills and team cohesion should be synonymous with a flattened hierarchy to enable effective care escalation.
