# Supplementary material for: Human factors in escalating acute ward care: a qualitative evidence synthesis
Source: BMJ Open Qual. 2021 Feb 26;10(1):e001145. doi: 10.1136/bmjoq-2020-001145 (PMC7919590; doi:10.1136/bmjoq-2020-001145)
Supplement: Supplementary data [file bmjoq-2020-001145supp002.pdf]

1. HOSPITALIZATION/

2. TERTIARY CARE CENTERS/

3. (ward or wards).ab,ti.

4. (inhospital or inpatient* or "in hospital").ab,ti.

5. (hospitalised or hospitalized).ab,ti.

6. "general hospital".ab,ti.

7. "nurs* staff* ".ab,ti.

8. "in patient".ab,ti.

9. bedside.ab,ti.

10. outreach.ab,ti.

11. 1 or 2 or 3 or 4 or 5 or 6 or 7 or 8 or 9 or 10

12. FAILURE TO RESCUE,HEALTH CARE/

13. "fail* to rescue ".ab,ti.

14. VITAL SIGNS/

15. MONITORING,PHYSIOLOGIC/

16. "vital sign* ".ab,ti.

17. (track and trigger).ab,ti.

18."early warning".ab,ti.

19. "warning score* ".ab,ti.

20. "early sign* ".ab,ti.

21. "warning system* ".ab,ti.

22. (deteriorat* or escalat*).ab,ti.

23. triggering.ab,ti.

24. HOSPITAL RAPID RESPONSE TEAM/

25. "rapid response".ab,ti.

26. "critical care outreach".ab,ti.

27. 12 or 13 or 14 or 15 or 16 or 17 or 18 or 19 or 20 or 21 or 22 or 23 or 24 or 25 or 26

28. MEDICAL ERROR/

29. DELAYED DIAGNOSIS/

30. COMMUNICATION/

31. PATIENT CARE TEAMS/

32. PATIENT SAFETY/

33. ORGANIZATIONAL CULTURE/

34. LEADERSHIP/

35. "human factor* ".af.

36. "human error* ".af.

37. "clinical error* ".af.

38. "medical error* ".af.

39. "protocol adherence".af.

40. "protocol compliance".af.

41. "teamwork*".af.

42. communication.af.

43. ("socio cultural" or sociocultural).af.

44. "situation awareness".af.

45. "organisational culture".af.

46. "organizational culture".af.

47. "safety culture".af.

48. "patient safety".af.

49. leadership.af.

50. "root cause analysis".af.

51. 28 or 29 or 30 or 31 or 32 or 33 or 34 or 35 or 36 or 37 or 38 or 39 or 40 or 41 or 42 or 43 or 44 or 45 or 46 or 47 or 48 or 49 or 50

52. 11 and 27 and 51
