# Supplementary figures and images for: Human factors in escalating acute ward care: a qualitative evidence synthesis
Source: BMJ Open Qual. 2021 Feb 26;10(1):e001145. doi: 10.1136/bmjoq-2020-001145 (PMC7919590; doi:10.1136/bmjoq-2020-001145)

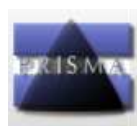

### Supplementary File 3 PRISMA 2009 Flow Diagram

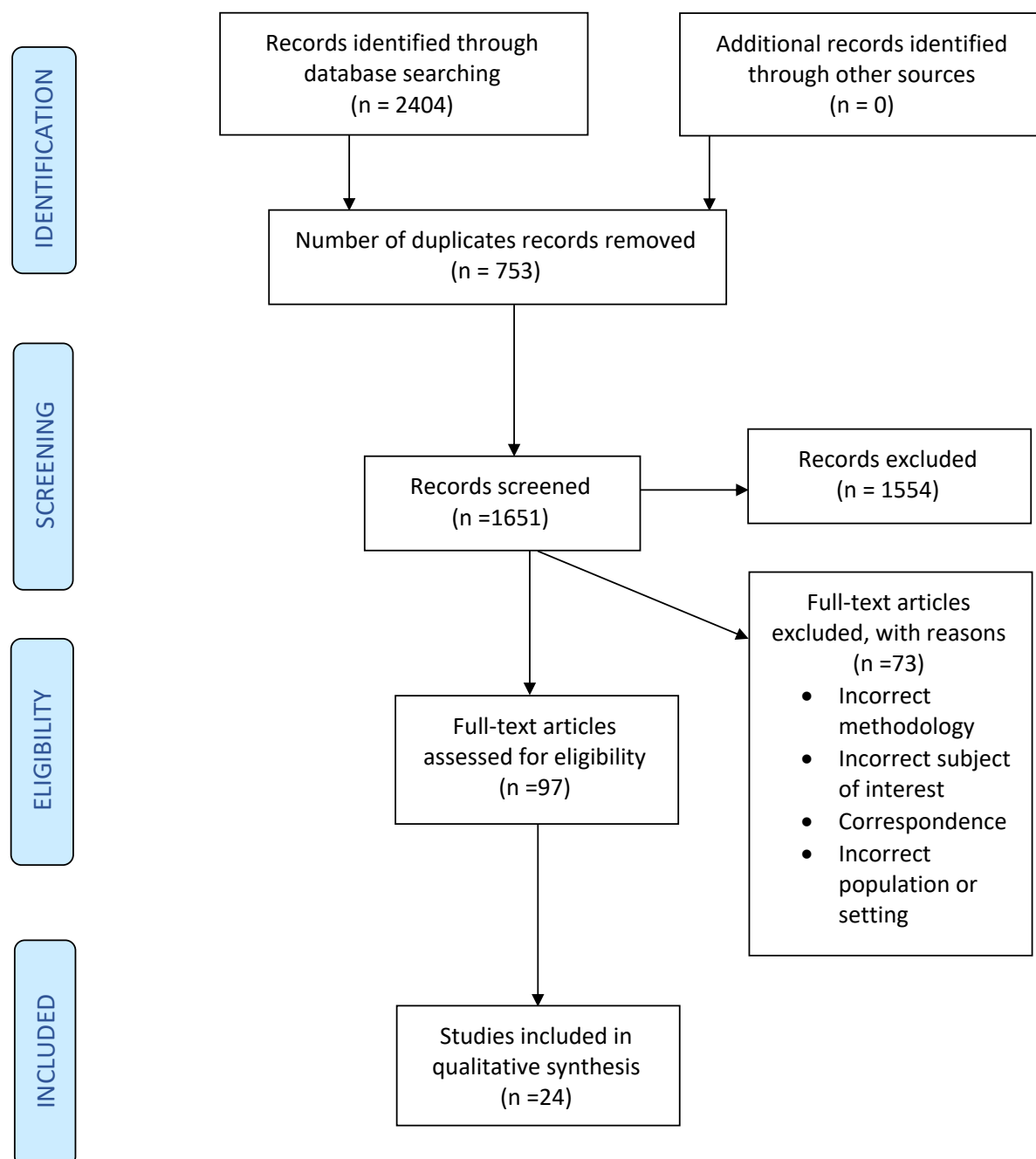

Supplement: Supplementary data [file bmjoq-2020-001145supp003.pdf]

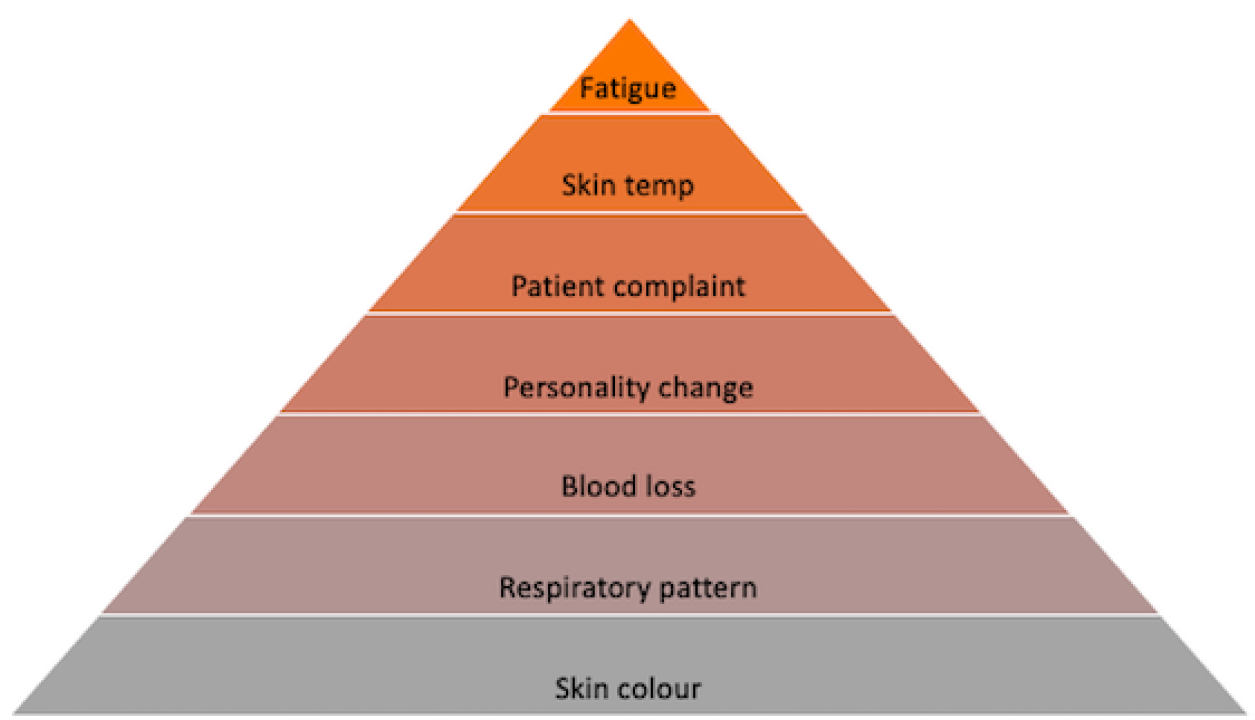

Supplement: Supplementary data [file bmjoq-2020-001145supp004.pdf]
